# Modulation of T cell function through L-arginine metabolism: a new therapy from an old enemy

**DOI:** 10.1186/2051-1426-1-S1-O10

**Published:** 2013-11-07

**Authors:** Matthew Fletcher, Maria E Ramirez, Rosa Sierra, Patrick Raber, Paulo Rodriguez

**Affiliations:** 1 Stanley S. Scott Cancer Center, Louisiana State University Health Sciences Center, New Orleans, LA, USA; 2 Department of Microbiology, Immunology and Parasitology, Louisiana State University Health Sciences Center, New Orleans, LA, USA; 3 Department of Pediatrics, Louisiana State University Health Sciences Center, New Orleans, LA, USA

## 

Recent studies have suggested the relevance of different energy metabolic pathways in the balance between protective T cell immunity and T cell anergy in tumors. We and others have suggested the role of the depletion of the non-essential amino acid L-arginine as a mechanism for the induction of T cell suppression in tumors. Therefore, we hypothesize that it is possible to metabolically regulate T cell responses simply through the modulation of L-arginine. In this study, we aimed to determine the effect of a pegylated form of the human L-arginine-metabolizing enzyme arginase I (peg-Arg I) in T cell responses. Activation of antigen-specific CD4+ and CD8+ T cells in the presence of peg-Arg I prevented cell proliferation and production of IFNγ in vitro and in vivo. Similarly, peg-Arg I impaired proliferation and IFNγ production in T cells activated with PMA/Ionomycin, suggesting that the effect of peg-Arg I was independent of T cell receptor (TCR) signaling. In fact, the anti-proliferative effect induced by peg-Arg I correlated with an arrest of T cells in the G0-G1 phase of the cell cycle, a decreased expression of cyclin D3 and cdk4, and a major inhibition of de novo translation. Interestingly, treatment of T cells with peg-Arg I did not impair the expression of activation markers CD25, CD69, and the production of IL-2, which correlated with an intact mitochondrial biogenesis. As a result, peg-Arg I did not have an effect in oxygen consumption (OCR) by mitochondrial respiration, but significantly blocked glycolytic pathways in activated T cells. Furthermore, peg-Arg I treated T cells increased the expression of genes associated with integrated stress responses (IRS) and arrest in translation including GCN2, Chop, and Atf4. In fact, GCN2 was a major mediator of the effects induced by peg-Arg I. Then, we tested the effect of peg-Arg I in mouse models of graft versus host disease (GVHD) and inflammatory bowel disease (IBD), both mediated through activated T cells. Peg-Arg I significantly extended the survival of mice in these 2 disease models, which associated with a decreased production of IFNγ. Altogether the results suggest the potential effect of the modulation of the metabolism of L-arginine as a mean to modulate T cell responses. Continuation of this study will advance in the understanding of the metabolic effects of L-arginine in T cell function, which could enable the development of therapies to modulate T cell responses in transplantation or autoimmunity.

